# Supplementary material for: Multi-client distributed blind quantum computation with the Qline architecture
Source: Nat Commun. 2023 Nov 25;14:7743. doi: 10.1038/s41467-023-43617-0 (PMC10676426; doi:10.1038/s41467-023-43617-0)
Supplement: Supplementary file 1 — Supplementary Information [file 41467_2023_43617_MOESM1_ESM.pdf]

Supplementary Information: Multi-client distributed blind quantum computation with the Qline architecture

Beatrice Polacchi,[1] Dominik Leichtle,[2] Leonardo Limongi,[1] Gonzalo Carvacho,[1] Giorgio Milani,[1] Nicolò Spagnolo,[1] Marc Kaplan,[3,*] Fabio Sciarrino,[1,†] and Elham Kashefi[2,4,5,‡]

[1]*Dipartimento di Fisica - Sapienza Università di Roma, P.le Aldo Moro 5, I-00185 Roma, Italy*
[2]*Laboratoire d'Informatique de Paris 6, CNRS, Sorbonne Université, 75005 Paris, France*
[3]*VeriQloud, 13 rue Victor Hugo, 92 120 Montrouge, France*
[4]*School of Informatics, University of Edinburgh, 10 Crichton Street, EH8 9AB Edinburgh, United Kingdom*
[5]*National Quantum Computing Centre, Didcot, OX11 0QX, U.K.*

SUPPLEMENTARY NOTE 1 - SECURITY ANALYSIS

In this section, we provide the details of the security proof for the full multi-client blind quantum computation protocol. The security analysis uses techniques and results from previous work on the Qline architecture [1], on quantum secure multi-party computing (QSMPC) [2], and on blind delegated computing using trusted Remote State Rotation (RSR) [3].

A. Abstract Cryptography

The security proof is given in the Abstract Cryptography (AC) framework [4]. The AC framework is used to define cryptographic protocols, and analyze their security. As opposed to stand-alone and game-based security models, it allows the secure composition of protocols within the framework. In this sense, if two or more protocols have been proven to be AC-secure, so is their sequential or parallel composition.

In the following, we refer to protocols, usually denoted by π, and resources, which we will denote by $\mathcal{R}$. Protocols can be used to construct new resources from other resources, denoted $\mathcal{R}_1 = \pi\mathcal{R}_0$. In order to define security for such constructions, one requires a sense of distance and equivalence in the space of resources. This is captured by the following notion of indistinguishability.

Definition 1 (Indistinguishability of resources)**.** *Let $\mathcal{R}_0$, $\mathcal{R}_1$ be resources with matching input and output interfaces. They are called ε-(statistically)-indistinguishable, denoted $\mathcal{R}_0 \approx_\varepsilon \mathcal{R}_1$, if for all (unbounded) distinguishers $\mathcal{D}$ it holds that*

$$|\Pr[b = 1 | b \leftarrow \mathcal{D}\mathcal{R}_0] - \Pr[b = 1 | b \leftarrow \mathcal{D}\mathcal{R}_1]| \leq \varepsilon.$$

Equipped with the pseudo-metric induced by the indistinguishability of resources, we can now proceed to define what it means to securely construct a resource, even in the presence of malicious parties, i.e., parties that deviate from the instructions specified by the protocol.

Definition 2 (Secure construction of resources)**.** *Let π be an n-party protocol, and let $P \subseteq \mathfrak{P}(\{1,\ldots,N\})$. π is then said to ε-securely construct resource $\mathcal{R}_1$ from resource $\mathcal{R}_0$ against adversarial patterns P if the following two properties hold:*

1. *Correctness: $\pi\mathcal{R}_0 \approx_\varepsilon \mathcal{R}_1 \perp$, where $\perp$ is used to filter all malicious interfaces;*
2. *Soundness: for all subsets $M \in P$ of colluding corrupted parties, there exists a simulator σ_M such that $\pi_{M^c}\mathcal{R}_0 \approx_\epsilon \mathcal{R}_1\sigma_M$.*

As mentioned previously, one power of the AC framework lies in the secure composability of resources. The following theorem [4, Theorem 1] is the formalization of this claim.

* kaplan@veriqloud.fr
† fabio.sciarrino@uniroma1.it
‡ elham.kashefi@lip6.fr

Theorem 1 (General composition of resources)**.** *Let $\mathcal{R}_0, \mathcal{R}_1, \mathcal{R}_2$ be resources, π_0, π_1 be protocols, and* id *be the identity protocol. Let $\circ$ denote the sequential composition, and $|$ denote the parallel composition of protocols and resources, respectively. The following statements then hold:*

1. *Sequential composition: $\pi_0\mathcal{R}_0 \approx_{\varepsilon_0} \mathcal{R}_1$ and $\pi_1\mathcal{R}_1 \approx_{\varepsilon_1} \mathcal{R}_2$ imply $(\pi_1 \circ \pi_0)\mathcal{R}_0 \approx_{\varepsilon_0+\varepsilon_1} \mathcal{R}_2$;*
2. *Context-insensitivity: $\pi_0\mathcal{R}_0 \approx_{\varepsilon} \mathcal{R}_1$ implies $(\pi_0|\mathsf{id})(\mathcal{R}_0|\mathcal{R}_2) \approx_{\varepsilon} (\mathcal{R}_1|\mathcal{R}_2)$.*

Note that the combination of sequential composition and context-insensitivity yields the security of parallel composition as well.

B. Ideal functionalities

In the AC framework, the desired, perfect behavior of protocols is captured by resources that we call ideal functionalities. We present the ideal functionalities of the resources relevant to the presented security proof in the following.

Supplementary Resource 1 describes the *Remote State Rotation* functionality, as defined in [3, Definition 7]. Its purpose is to capture the quantum abilities of the clients which are limited to performing single-qubit rotations around the z-axis.

Supplementary Resource 1: Remote State Rotation

Inputs:

- The client sends an angle $\theta \in \mathcal{A} = \{\frac{j\pi}{4} | j = 0, \ldots, 7\}$.
- The server sends a single-qubit quantum state ρ.

Computation by the resource:

1. Set $\rho' = R_z(\theta)\rho(R_z(\theta))^\dagger$ and return ρ' to the server.

Supplementary Resource 2 captures *Blind Delegated Quantum Computing* without verifiability [5]. In our version of the ideal functionality, we restrict the delegated computation to classical inputs. A malicious server (setting $c = 1$) has the option to corrupt the state that the client receives at the end of the protocol.

Supplementary Resource 2: Blind Delegated Quantum Computing

Inputs:

- The client sends a classical bit string x.
- The server sends a flag $c \in \{0, 1\}$. If $c = 1$, the server also sends a quantum state ψ, and the description of a map $\mathcal{E}$.

Computation by the resource:

1. If $c = 0$, the resource computes the correct output $y = \mathcal{U}(|x\rangle\langle x|)$ and sends it to the client.
2. If $c = 1$, the resource computes the corrupted output $y = \mathcal{E}(|x\rangle\langle x| \otimes \psi)$ and sends it to the client.

Note that universal computation is possible with Supplementary Resource 2 if $\mathcal{U}$ is a universal quantum map, and the description of the target quantum computation is encoded in the client's input x. For honest servers, the filter $\perp_{c=0}$ sets $c = 0$ and blocks access to the other interface on the server's side.

Finally, we need to describe the ideal functionality of our target Supplementary Resource 3, *Multi-Client Blind Quantum Computation.* As before, the jointly evaluated computations are restricted to classical inputs.

C. Security of the full protocol

Universal Blind Quantum Computing [6] is a protocol that allows a client to delegate a computation to a server with the guarantee that the server cannot learn anything about the computation, its inputs, or its outputs, except for the amount of resources that were used. In other words, only the computation graph and the order of qubits are leaked to the server. Supplementary Protocol 1 is a version of this protocol that does not necessarily require universal resource states, but rather works for any graph that is usable to implement the target computation. In this

Supplementary Resource 3: Multi-Client Blind Quantum Computation

Inputs:

- For $j = 1, \dots, n$, client j sends a classical bit string x_j. It also inputs $c_j \in \{0,1\}$ as a filtered interface.
- The server inputs a flag $c \in \{0,1\}$.
- All malicious parties, that is all clients with $c_j = 1$, and the server if $c = 1$, jointly send a quantum state ψ and the description of a map $\mathcal{E}$.

Computation by the resource:

1. If $c_j = c = 0$ for all $j = 1, \dots, n$, the resource computes the correct output $y = \mathcal{U}\left(\bigotimes_{j=1}^n |x_j\rangle\langle x_j|\right)$ and sends it to the clients.
2. Otherwise, the resource computes the corrupted output $y = \mathcal{E}\left(\bigotimes_{j=1}^n |x_j\rangle\langle x_j| \otimes \psi\right)$ and sends it to the clients.

way, clients can opt to use cheaper, non-universal resource states that are closer to the actual requirements of the target algorithm rather than using more expensive universal resource states. This however will leak some additional information to the server as it restricts the class of algorithms that could potentially be performed using the chosen resource state. Of course, it remains possible to use universal resource states for full blindness.

Supplementary Protocol 1: Blind Quantum Computation

Public Information:

- A graph $G = (V, E, I, O)$ with input and output vertices I and O, respectively.
- A partial order $\preceq$ on the set V of vertices.

Inputs:

- The client has as input a classical bit string $x \in \{0,1\}^{|I|}$. It further inputs a set of angles $(\phi_v)_{v \in V}$, and a flow f on G compatible with $\preceq$.
- The server has no inputs.

Protocol:

1. For all $v \in V$, the client samples an angle $\theta(v) \leftarrow_R \mathcal{A}$ uniformly at random, prepares the single-qubit state $|+_{\theta(v)}\rangle$, and sends it to the server.
2. The server applies the entangling operation according to the graph G, i.e., for every edge $\{v, w\} \in E$, the server applies the two-qubit gate CZ_{vw}.
3. For all $v \in V \setminus O$, such that the partial order $\preceq$ is respected, the client and the server perform the following interactive steps:
   (a) The client uses the previous (corrected) measurement outcomes to compute the corrected angle $\phi'(v)$ from $\phi(v)$ according to the flow f.
   (b) The clients samples a bit $r(v) \leftarrow_R \{0,1\}$ uniformly at random, calculates $\delta(v) = \phi'(v) + \theta(v) + (r(v) + x(v))\pi$, and sends $\delta(v)$ to the server.
   (c) The server measures qubit v in the $|\pm_{\delta(v)}\rangle$-basis and returns the measurement outcome $m(v)$ to the client.
   (d) The client calculates the corrected measurement outcome as $m'(v) = m(v) \oplus r(v)$.
4. The client outputs the bit string $(m'(v))_{v \in V \setminus O}$. The server further sends the qubits in O to the client, who decrypts them (using $\theta(v)$, $r(v)$ and $m'(v)$ as keys) and keeps them as additional output.

Previous work showed that Supplementary Resource 1 can be used in combination with Supplementary Protocol 1 to securely construct Supplementary Resource 2. Since we use this result as part of our security proof, we reiterate it here for convenience [3, Theorem 4].

Theorem 2. *The BQC Supplementary Protocol 1 where the client uses Supplementary Resource 1 to remotely prepare the required single-qubit states perfectly constructs Supplementary Resource 2 against a malicious server.*

The security analysis of the multi-client protocol proposed in this work follows a modular paradigm. In this spirit, we first analyze the security of Supplementary Protocol 2, the subprotocol which consists of the communication of a single qubit along the Qline. Since both the photon source and the server are untrusted and potentially colluding in the protocol, they are treated as a single untrusted party in the following.

Supplementary Protocol 2: Collaborative Remote State Rotation

Inputs:

- The n clients have no input.
- The orchestrator has as input an angle $\theta \in \mathcal{A}$.
- The server receives as input a single-qubit quantum state ρ.

Protocol:

1. For $j = 1, \ldots, n$, client j samples uniformly at random $\theta_j \leftarrow_R \mathcal{A}$, and sends θ_j to the orchestrator.
2. The server sends ρ to client 1.
3. For $j = 1, \ldots, n$, client j applies the operation $R_z(\theta_j)$ to the received quantum state and forwards it to the next client. After applying its own rotation, client n forwards the final state to the server.
4. The orchestrator computes $\theta' = \theta - \sum_{j=1}^{n} \theta_j \pmod{2\pi}$ and sends θ' to the server.
5. The server applies the operation $R_z(\theta')$ to the single-qubit state that it received from client n, and keeps the resulting state as its output.

We continue to prove the security of Supplementary Protocol 2.

Theorem 3. *Supplementary Protocol 2 perfectly constructs Supplementary Resource 1 between the orchestrator and the server from secure classical and quantum channels against malicious coalitions of at most the server and $n-1$ clients.*

Proof of correctness. If all participating parties are acting honestly, the single-qubit quantum state sent from client n to the server takes the following form:

$$R_z(\bar{\theta})\rho \left(R_z(\bar{\theta})\right)^\dagger ,$$

where $\bar{\theta} = \sum_{j=1}^{n} \theta_j$. After the final correction which is applied to this state by the server, the output becomes

$$R_z(\theta')R_z(\bar{\theta})\rho \left(R_z(\bar{\theta})\right)^\dagger \left(R_z(\theta')\right)^\dagger = R_z(\theta)\rho \left(R_z(\theta)\right)^\dagger ,$$

which shows that the protocol is correct. □

Proof of soundness. As security in the AC framework is simulation-based, we need to provide the construction of a simulator fit to translate real-world to ideal-world attacks. In the following, we assume the worst case of a colluding malicious coalition of the server and $n-1$ clients. Because the protocol and the ideal resource are sufficiently symmetric in the enumeration of the clients, we can assume without loss of generality that the first client behaves honestly. The construction of the simulator for this scenario is given as Supplementary Simulator 1.

Supplementary Simulator 1: Malicious server and clients $2, \ldots, n$

Behavior of the simulator:

1. The simulator expects angles $\theta_j \in \mathcal{A}$ for $j = 2, \ldots, n$ from the malicious clients, and a single-qubit quantum state ρ from the malicious server.
2. The simulator forwards ρ to the ideal functionality described by Supplementary Resource 1, and receives the state ρ' from it.
3. It samples uniformly at random the angle $\theta_1 \leftarrow_R \mathcal{A}$.
4. It applies the operation $R_z(\theta_1)$ to ρ' and returns the resulting quantum state to the malicious server.
5. Finally, the simulator computes $\theta' = -\sum_{j=1}^{n} \theta_j$ and sends θ' to the malicious server.

It remains to be shown that the views of the distinguisher in the real world where it has access to the inputs θ, ρ and to the views of all malicious parties, and in the ideal world where it has access to the inputs θ, ρ and to the interfaces to the simulator are perfectly equal. These two views can be summarized as follows:

	**Real world**	**Ideal world**
Input angle	θ	θ
Input state	ρ	ρ
Client 1 output	$R_z(\theta_1)\rho(R_z(\theta_1))^\dagger$	$R_z(\theta+\theta_1)\rho(R_z(\theta+\theta_1))^\dagger$
Correction	$\theta - \sum_{j=1}^n \theta_j$	$-\sum_{j=1}^n \theta_j$

Since θ_1 is chosen uniformly at random by the simulator in the ideal world, we can substitute it by $\theta_1 - \theta$ without changing the view of the distinguisher. This yields:

**Real world**	**Ideal world**
θ	θ
ρ	ρ
$R_z(\theta_1)\rho(R_z(\theta_1))^\dagger$	$R_z(\theta_1)\rho(R_z(\theta_1))^\dagger$
$\theta - \sum_{j=1}^n \theta_j$	$\theta - \sum_{j=1}^n \theta_j$

Clearly, these two distributions are identical, which proves that the views of the distinguisher in the two worlds are perfectly indistinguishable. □

It now remains to piece together all building blocks to obtain the security of the full protocol.

Theorem 4. *The Multi-Client Blind Quantum Computation Protocol 1 of the main text, where the orchestrator is replaced by a classical SMPC resource between all other parties, perfectly constructs Supplementary Resource 3 against malicious coalitions of at most the server and $n-1$ clients.*

Proof. After having established the security of all building blocks, this proof is straightforward and works by repeated application of the general composition principle in Supplementary Theorem 1, analogously to the proof of [2, Theorem 5].

Retracing the steps of the construction of the protocol in question, we begin by replacing the classical SMPC resource by a trusted classical party, the orchestrator. This step does not incur any security loss.

Next, Supplementary Theorem 3 allows us to replace every execution of Supplementary Protocol 2 by one call to Supplementary Resource 1, with zero security loss.

Finally, Supplementary Theorem 2 establishes that the remaining protocol between orchestrator and server is indeed a secure construction of the Blind Delegated Quantum Computing Supplementary Resource 2. Keeping in mind that the orchestrator additionally is in charge of collecting the clients' inputs and distributing the outcome of the computation, this protocol is indeed a perfect realization of Supplementary Resource 3. □

Remark 1 (Removing redundant correction steps)**.** *When considering the realization of a resource by itself, the final correction step in Supplementary Protocol 2 is indeed necessary, as this is the only way for the simulator to transmit the correct quantum state to the server. However, when using the protocol in the context of UBQC, this correction step can be combined with the corrections that are anyway present in the BQC protocol, and do hence not need to be performed as a separate round of communication. This observation is similar to the one made by [2] in the context of Collaborative RSP and QSMPC.*

Remark 2 (Varying the location of the entangling step)**.** *The security of the protocol is independent of whether the qubits are entangled before or after their communication along the Qline. Therefore, both setups in which the photon source creates cluster states and the server performs only adaptive measurements, as well as setups in which the photon source emits unentangled qubits, and entanglement and measurements are performed by the server are possible. Even combinations of both, in which part of the entanglement is created before, and part after the Qline, are conceivable.*

SUPPLEMENTARY NOTE 2 - TIME SCHEME OF THE EXPERIMENTAL PROTOCOL

At the beginning of each round of the protocol, the clients Alice and Bob set their liquid crystals (LCs) according to the phase shifts they want to apply to their qubits (the θ_i^j random parameters) and communicate all their secret parameters to the system dedicated to the TTP. With this information, the TTP computes the measurement angle for the first photon according to the formula δ_1, and it pre-computes the two possible values for δ_2, given that the outcome of the first measurement is still unknown, namely $\delta_2^{\pm} = \theta_2 + x_2\pi + r_2\pi \pm \phi_2$. These angles correspond to two possible voltages, either V_1 or V_2. This stage is necessary to keep the time delay between the two photons reasonable, and so the losses. Indeed, while the first round of classical communication would take time intervals of the order of the milliseconds (since one would need to wait for all the settings to be performed), the communication of the

measurement outcome alone takes, instead, some tens of nanoseconds only, since it happens through analog signals. $\approx$ ms time-delays would need hundreds of km long-fibers, while $\approx$ ns time-delays only needs some meters long fibers. The first measurement angle δ_1 is communicated to S_2. Starting from time t_1, the quantum part of the experiment begins. The photon pairs are generated and sent through Alice's and Bob's LCs, and S_2 measures the first qubit. The outcome of this measurement, m_1, is communicated to the TTP. Starting from t_2, the TTP can communicate the measurement angle δ_2 to S_2 through a voltage that activates suitably the Pockels cell, and so the second photon is measured. In the end, the outcome of this measurement is sent to the TTP which communicates the final result of the measurement to the clients, namely $m_2^{true} = m_2 \oplus r_2$.

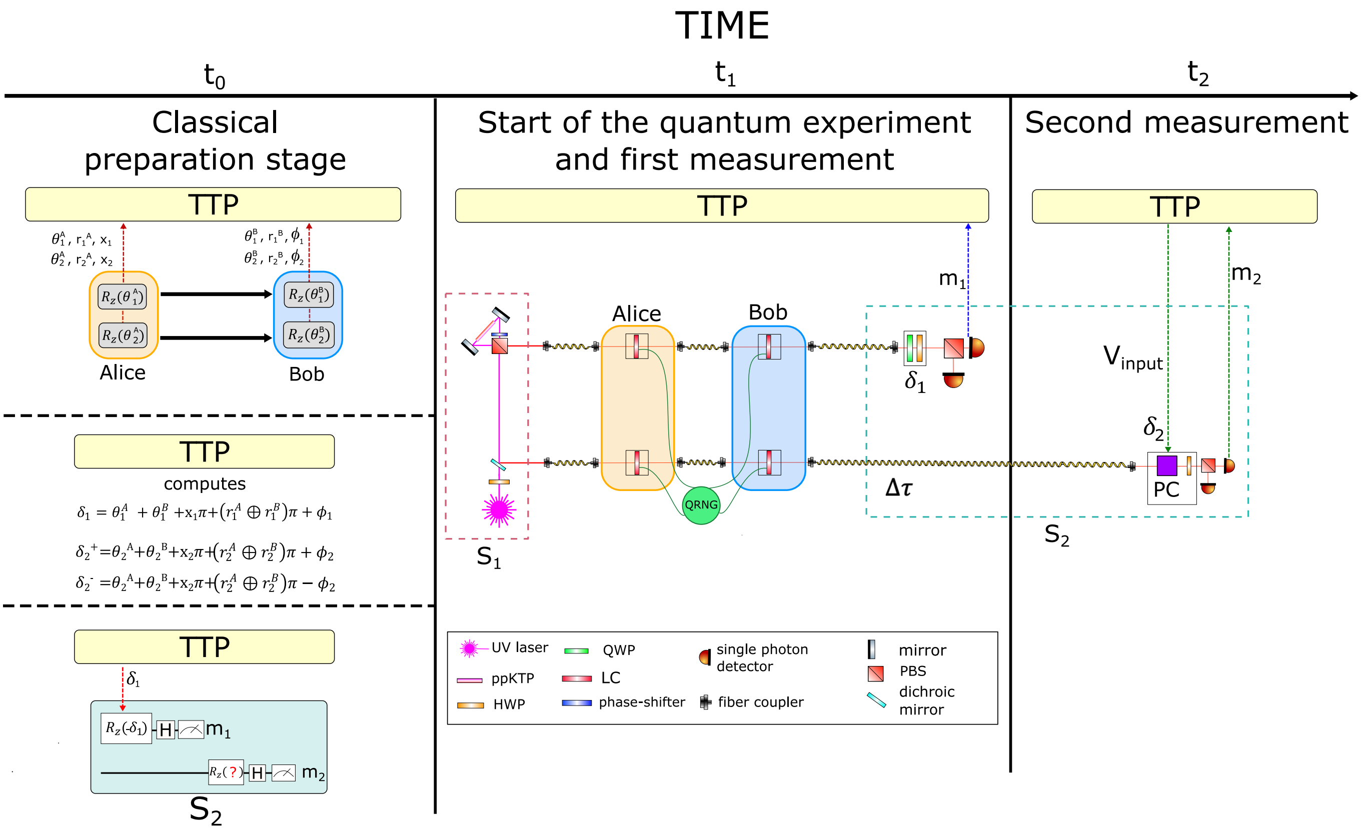

Supplementary Figure 1: **Time scheme of the experiment.** Here, we depict the time scheme of the experiment. It is divided into three main stages: (t_0) the classical preparation stage, where the clients set up their liquid crystals and communicate their secret parameters to the TTP, (t_1) the start of the quantum part of the protocol and the first measurement, (t_2) third and last round where the second measurement happens. The second photon is delayed through a ≈ 65 m long single-mode fiber.

SUPPLEMENTARY NOTE 3 - STATE PREPARATION AND MEASUREMENT

We encode the computational basis in the polarization of photons, namely $|0\rangle = |H\rangle$ and $|1\rangle = |V\rangle$. We generate entangled pairs of photons in the state $\frac{1}{\sqrt{2}}(|HH\rangle + |VV\rangle)$ and then suitably rotate the polarization of the second photon to obtain the state $\frac{1}{2}(|HH\rangle + |HV\rangle + |VH\rangle - |VV\rangle)$.

Afterward, the clients rotate their photons in the desired state, namely, by randomly choosing the parameters $\theta_1^{\rm A}$, $\theta_2^{\rm A}$, $\theta_1^{\rm B}$, $\theta_2^{\rm B}$, from the set $\{0, \ \pi/4, ..., \ 7\pi/4\}$, and by manipulating its polarization through a sequence of liquid crystals, whose action can be described by the following operator:

$$R_z(\theta) = \begin{pmatrix} 1 & 0 \\ 0 & e^{i\theta} \end{pmatrix} \tag{1}$$

Projections in the basis $\{|+_{\delta_1}\rangle, |-_{\delta_1}\rangle\}$ are performed by use of a quarter-wave plate rotated at angle $-\pi/4$ with

respect to its optic axis, followed by a half-wave plate (HWP) rotated at angle $\pi/8 + \delta_1/4$ with respect to its optic axis, and a polarizing beam splitter (PBS).

$$O(\delta_1) = U^\dagger_{QWP}(-\pi/4)\ U^\dagger_{HWP}(\pi/8 + \delta_1/4)\ O_{PBS}\ U_{HWP}(\pi/8 + \delta_1/4)\ U_{QWP}(-\pi/4) \tag{2}$$

Projections in the basis $\{|+_{\delta_2}\rangle, |-_{\delta_2}\rangle\}$ are performed by use of a Pockels cell which is activated with a suitable voltage, corresponding to a given phase ϕ, followed by a HWP rotated at angle $\pi/8$ with respect to its optic axis, and a PBS, as described by Supplementary Equation (3).

In Supplementary Table I, we show the phase shifts necessary to implement the desired measurement $O(\delta_2)$. We optimize the number of different voltages needed, by applying an outcome flip (f) whenever the phase shift required is larger or equal to π. The resulting operator is the following:

$$O(\delta_2) = X^f\ U^\dagger_{PC}(\phi)\ U^\dagger_{HWP}(\pi/8)\ O_{PBS}\ U_{HWP}(\pi/8)\ U_{PC}(\phi)\ X^f \tag{3}$$

$\boldsymbol{\delta_2}$	**Ideal phase-shift**	**PC phase-shift**	**f**
0	0	0	0
$\pi/4$	$7\pi/4$	$3\pi/4$	1
$\pi/2$	$3\pi/2$	$\pi/2$	1
$3\pi/4$	$5\pi/4$	$\pi/4$	1
π	π	0	1
$5\pi/4$	$3\pi/4$	$3\pi/4$	0
$3\pi/2$	$\pi/2$	$\pi/2$	0
$7\pi/4$	$\pi/4$	$\pi/4$	0

Supplementary Table I

SUPPLEMENTARY NOTE 4 - SYSTEM FOR MEASUREMENT ADAPTIVITY

In order to experimentally implement the feed-forward system, i.e. measuring the first qubit and consequently choosing the measurement angle δ_2 according to the outcome m_1^{true}, it is necessary to introduce a temporal delay between the two photons' paths of ≈ 320 ns or ≈ 65 m in fiber. This time delay corresponds to the time needed to compute δ_2 and to feed the Pockels cell with a suitable voltage accordingly. Supplementary Table I shows that the eight possible values for δ_2 can be implemented by four independent rotations, performed by a Pockels cell, in addition to a final bit-flip ($f = 0, 1$) performed in post-processing by the server.

We designed a logic circuit that takes as input nine bits and produces a five-bit output, in formulas

$$g(A, B, r_1, m_1^+, m_1^-) = VT \tag{4}$$

Specifically, the three-bit inputs $A = a_2a_1a_0$ and $B = b_2b_1b_0 \in \{000, 001, \ldots, 111\}$ encode respectively the clients' chosen parameters $\theta_2 + x_2\pi + r_2\pi$ and $\phi_2 \in \{0, \frac{\pi}{4}, \ldots, \frac{7\pi}{4}\}$. The single-bit $r_1 = r_1^{\mathrm{A}} \oplus r_1^{\mathrm{B}}$ corrects the value of m_1 into m_1^{true}. The two-bit input $\{m_1^+,\ m_1^-\} \in \{10, 01\}$ represents the only interesting physical output of the two detectors in the server's first measurement station encoding respectively $m_1 = 0, 1$. We define the function g such that, if $m_1^{true} = 0(1)$, then $\delta_2 = A + (-)B$. Thus, according to the theory, the inputs $r_1,\ m_1^+,\ m_1^-$ let the device choose if either to perform the sum or difference, both modulo eight, of the inputs $A,\ B$, that corresponds to evaluating the angle δ_2. Instead, the three-bit output $V = v_2v_1v_0$ encodes the evaluated δ_2 angle, while the output bits $m_1^{+true},\ m_1^{-true} \in \{10, 01\}$ encode respectively $m_1^{true} = 0, 1$. A scheme of our device is shown in Supplementary Figure 2. Our encoding values for $A,\ B,\ f,\ V$ are shown in the Supplementary Table II, in addition to the association between the output fV and the required Pockels cell voltage V_i for $i = 0, \frac{\pi}{4}, \frac{\pi}{2}, \frac{3\pi}{4}$.

We now demonstrate that such a circuit would not need to be changed in its structure when having n clients. In our protocol, to ensure the privacy of all clients, each of them needs to rotate all the qubits involved in the computation. Then, one would first have to compute the cumulative secret rotation angle θ_i for the i-th qubit as $\theta_i = \sum_{j=1,\ldots,n} \theta_i^{C_j}$ and the cumulative random bit $r_i = \bigoplus_{j=1,\ldots,n} r_i^{C_j}$, where j indicates the j-th client. The classical input data x_2 and algorithm details ϕ_2 can be summed up analogously depending on the subset of clients that provided such information. Such cumulative parameters can then be placed in the coefficients A and B for the computation of the blind measurement angle δ_2. The remainder of the computation would not change.

$a_2a_1a_0 \leftrightarrow A$[rad]		$b_2b_1b_0 \leftrightarrow B$[rad]		$\delta_2 \leftrightarrow$	$f v_2 v_1 v_0$	$\leftrightarrow$**PC voltage**
000	0	000	0	0	0000	V_0
001	$\pi/4$	001	$\pi/4$	$\pi/4$	1100	$V_{3\pi/4}$
010	$\pi/2$	010	$\pi/2$	$\pi/2$	1010	$V_{\pi/2}$
011	$3\pi/4$	011	$3\pi/4$	$3\pi/4$	1001	$V_{\pi/4}$
100	π	100	π	π	1000	V_0
101	$5\pi/4$	101	$5\pi/4$	$5\pi/4$	0100	$V_{3\pi/4}$
110	$3\pi/2$	110	$3\pi/2$	$3\pi/2$	0010	$V_{\pi/2}$
111	$7\pi/4$	111	$7\pi/4$	$7\pi/4$	0001	$V_{\pi/4}$

Supplementary Table II

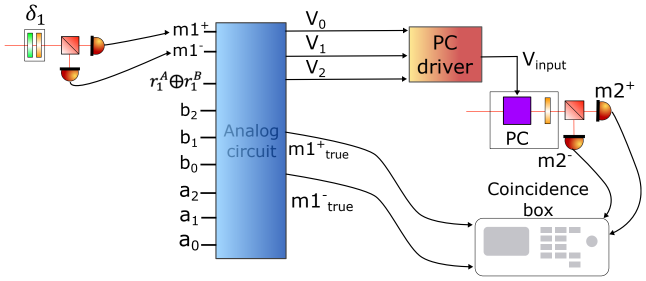

Supplementary Figure 2: **Fast data elaboration system.** A suitably programmed analog circuit gets as input: (i) three bits corresponding to the following angle $A = a_2a_1a_0 = (\theta_2 + x_2\pi + r_2\pi)\%(2\pi) \in \{0, ..., 7\pi/4\}$, (ii) three bits corresponding to $B = b_2b_1b_0 = \phi_2$, (iii) one bit equal to $r_1 = (r_1^{\mathrm{A}} \oplus r_1^{\mathrm{AB}})$, (iv) two signals corresponding to the outcome 0 and 1 of the first measurement, respectively m_1^+ and m_1^-. The function first corrects the outcome of the first measurement according to the formula: $m_1^{\pm true} = m1^{\pm} \oplus r_1$. Then, it computes the second measurement basis δ_2 and transforms it into a low-voltage pulse to be amplified and then applied to the Pockels cell. In detail, the outcome of the circuit is made up of: (i) three bits, V_0, V_1, V_2, corresponding to three different voltage amplification factors performed by the PC driver, (ii) a copy of the two corrected signals $m1^{\pm}_{true}$. The HV voltage pulse is sent to the PC and, after the second measurement is performed, its outcome is sent to a coincidence box as well as the first one. The second outcome is corrected in post-processing, by applying the formula $m2^{\pm}_{true} = m2^{\pm} \oplus f \oplus r_2$, where the values of f can be retrieved in Supplementary Table I.

SUPPLEMENTARY NOTE 5 - BLINDNESS OF THE FIRST QUBIT

Here, we report the details of the demonstration of the blindness of the first qubit. To show it, we fixed the measurement angle $\delta_2 = 3\pi/2$ and averaged the resulting density matrices of the first qubit over all possible clients' rotation angles θ_2^{A} and θ_2^{B}, namely 64 possible combinations. Here, as an instance, we report the quantum state tomographies for eight combinations, where Alice keeps her rotation angle fixed at $\theta_2^{\mathrm{A}} = 0$, while Bob varies it. The quantum state tomographies shown also confirm the correctness of the protocol in the case of the computation of a quantum function, taking the first qubit as the output of the computation. Indeed, each of these density matrices can be seen as the case where the computation angle amounts to $\phi_2 = \delta_2 - \theta_2^{\mathrm{A}} - \theta_2^{\mathrm{B}}$, where $\delta_2 = 3\pi/2$ and $\theta_2^{\mathrm{A}} = 0$.

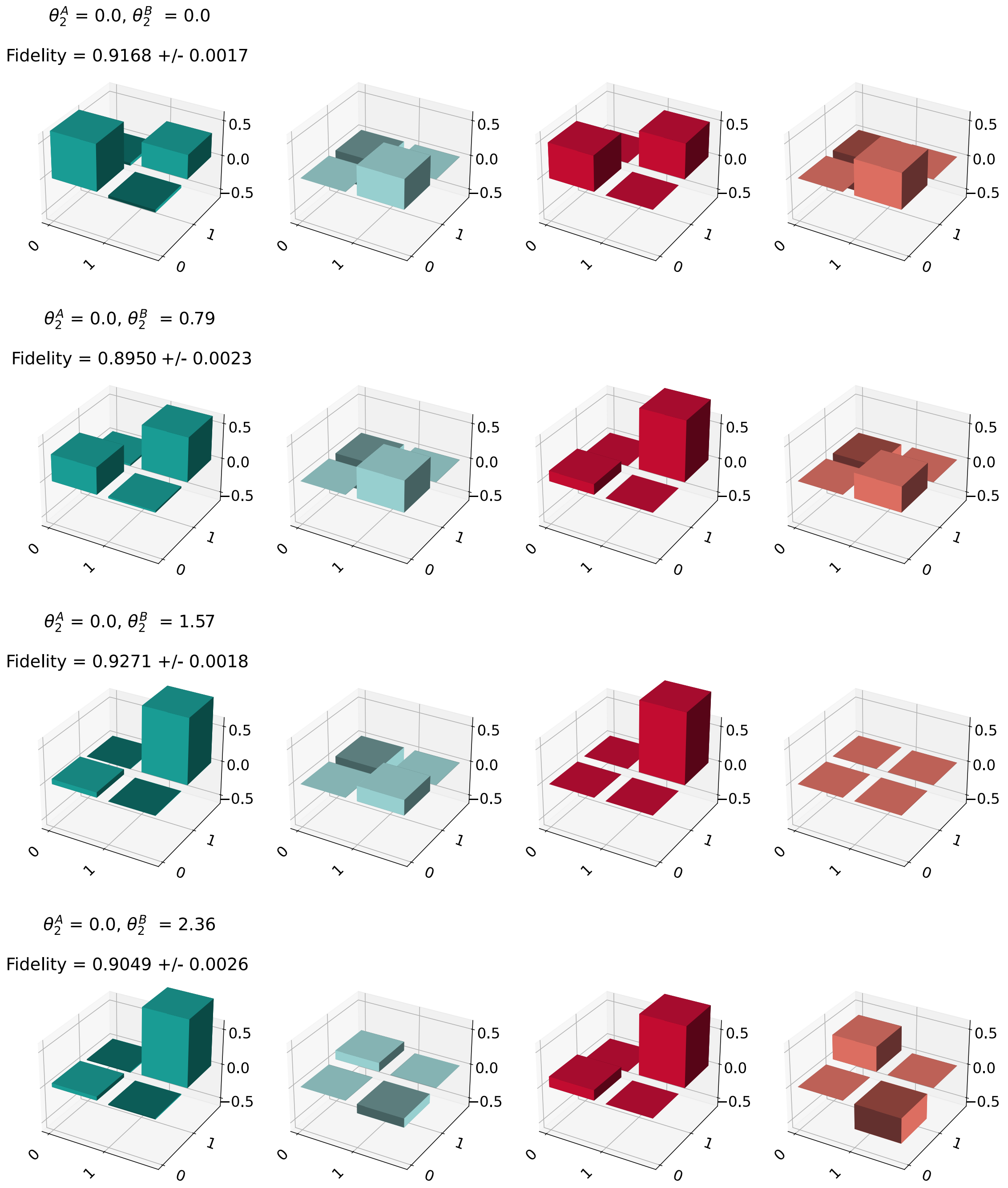
θ_2^A = 0.0, θ_2^B = 0.0
Fidelity = 0.9168 +/- 0.0017
θ_2^A = 0.0, θ_2^B = 0.79
Fidelity = 0.8950 +/- 0.0023
θ_2^A = 0.0, θ_2^B = 1.57
Fidelity = 0.9271 +/- 0.0018
θ_2^A = 0.0, θ_2^B = 2.36
Fidelity = 0.9049 +/- 0.0026

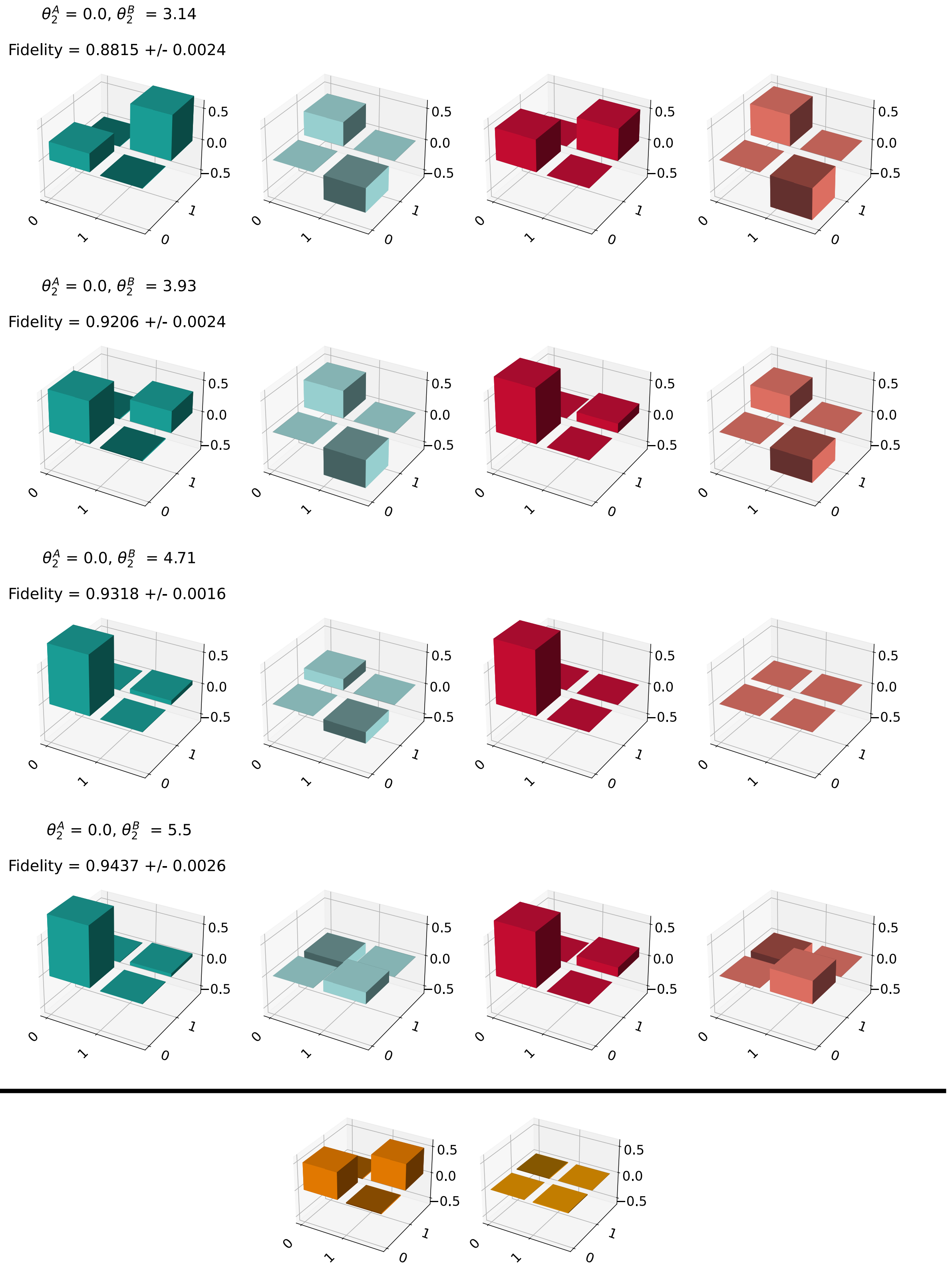

Supplementary Figure 3: **Experimental and theoretical quantum state tomographies to demonstrate blindness of the first qubit.** We show in blue and light blue, respectively, the real and imaginary parts of the experimental density matrix, and in red and light red the real and imaginary parts of the ideal density matrix. We also report the corresponding fidelities and the resulting completely mixed state. The fidelity with the completely mixed state amounts to $F_1 = 0.99949 \pm 0.00002$ and the Von Neumann entropy amounts to $S_1 = 0.99852 + / - 0.00006$.

SUPPLEMENTARY NOTE 6 - BLINDNESS OF THE SECOND QUBIT

In this section, we report the details of the demonstration of the blindness of the second qubit. To show it, we fixed the measurement angle $\delta_1 = \pi$ and averaged the resulting density matrices for the second qubit over all possible clients' rotation angles of the first qubit, namely 64 possible combinations. Here, as an instance, we report the quantum state tomographies for eight combinations and their comparison with the theoretical expectations, where Alice keeps her rotation angle fixed at $\theta_1^{\mathrm{A}} = \pi$, while Bob varies it. The quantum state tomographies shown also confirm the correctness of the protocol in the case of the computation of a quantum function. Indeed, each of these density matrices can be seen as the case where the first computation angle amounts to $\phi_1 = \delta_1 - \theta_1^{\mathrm{A}} - \theta_1^{\mathrm{B}}$, where $\delta_1 = \pi$ and $\theta_1^{\mathrm{A}} = \pi$.

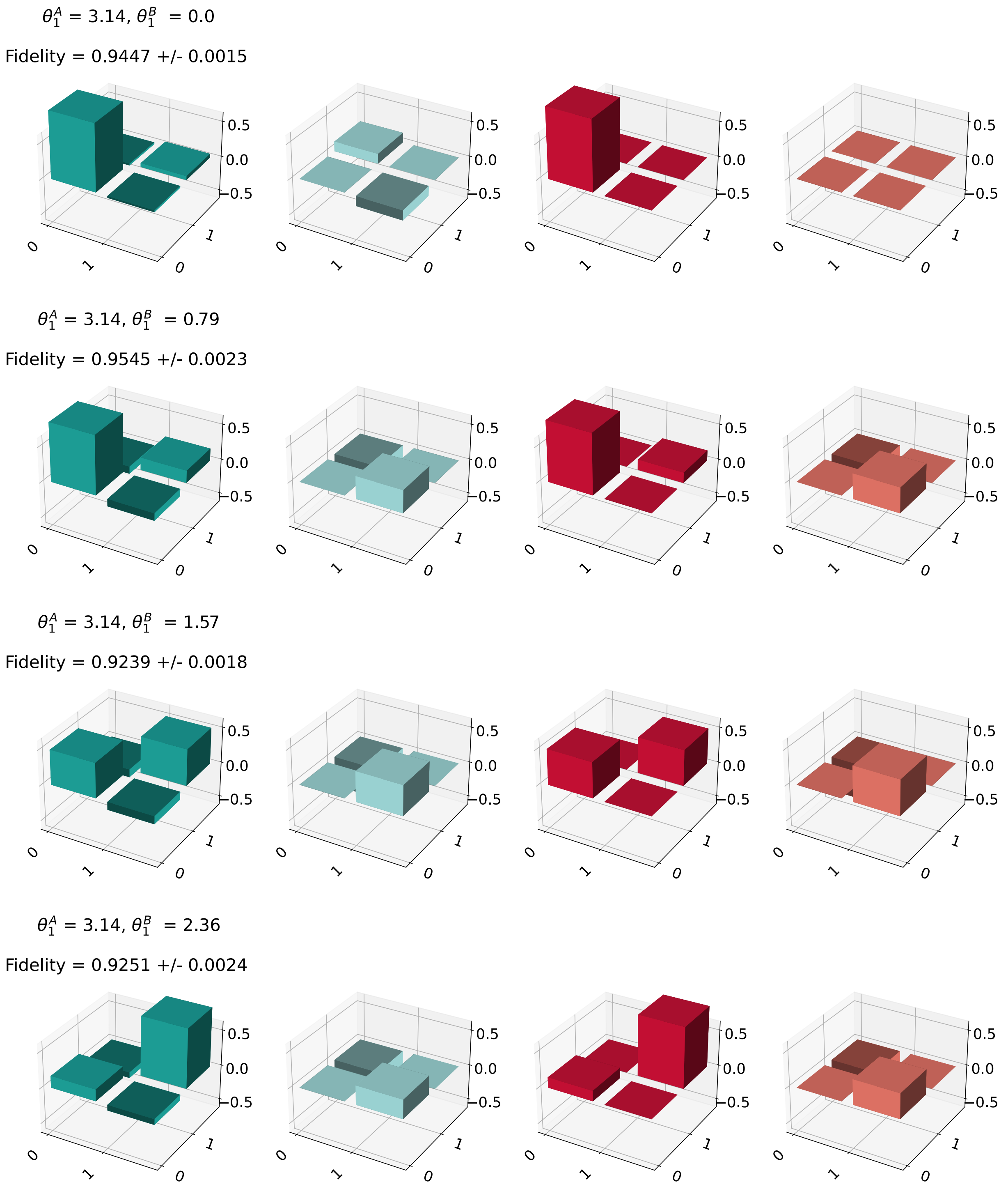

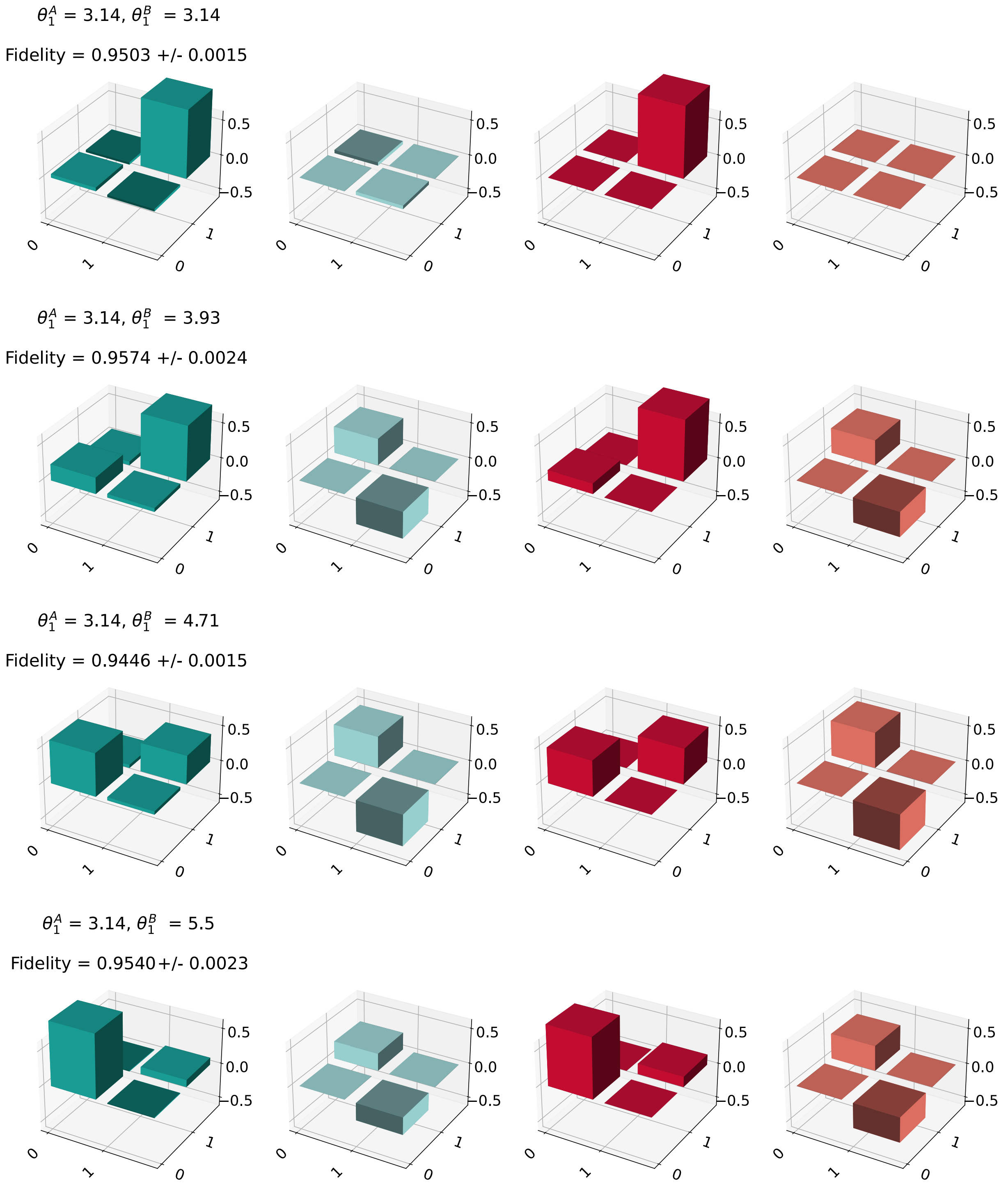

Supplementary Figure 4: **Experimental and theoretical quantum state tomographies to demonstrate blindness of the second qubit.** We show in blue and light blue, respectively, the real and imaginary parts of the experimental density matrix, and in red and light red the real and imaginary parts of the ideal density matrix. We also report the corresponding fidelities.

SUPPLEMENTARY NOTE 7 - BLINDNESS OF THE FULL INITIAL STATE

In this section, as an example, we report the quantum state tomographies for the first 16 combinations of the clients' parameters $\theta_1^{\mathrm{A}}, \theta_2^{\mathrm{B}}$ and the comparison with the theoretical expectations. We demonstrated the blindness of the full

initial state by averaging over all density matrices resulting from varying θ_1^{A}, θ_2^{B} in the set $\mathcal{A}$.

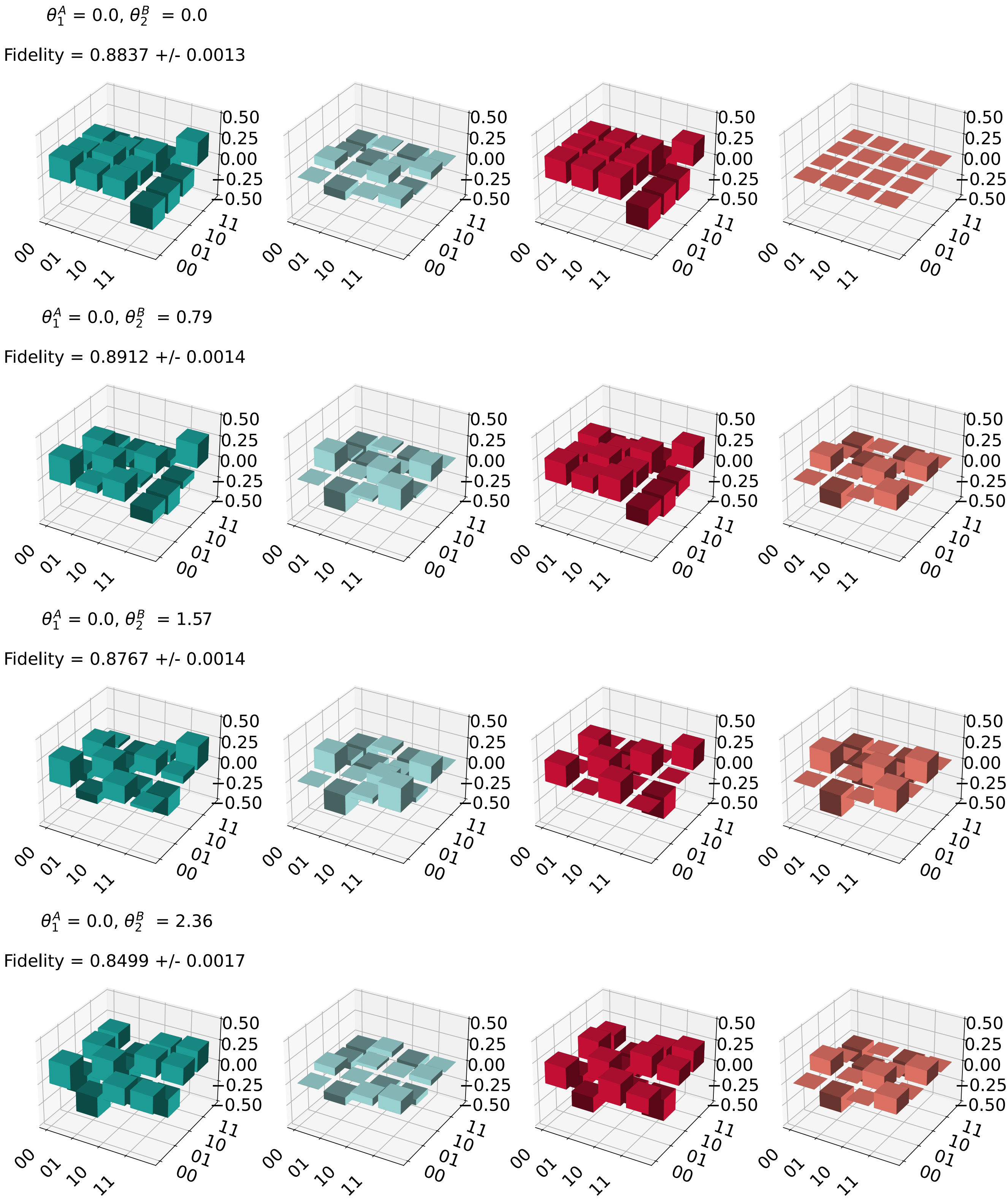

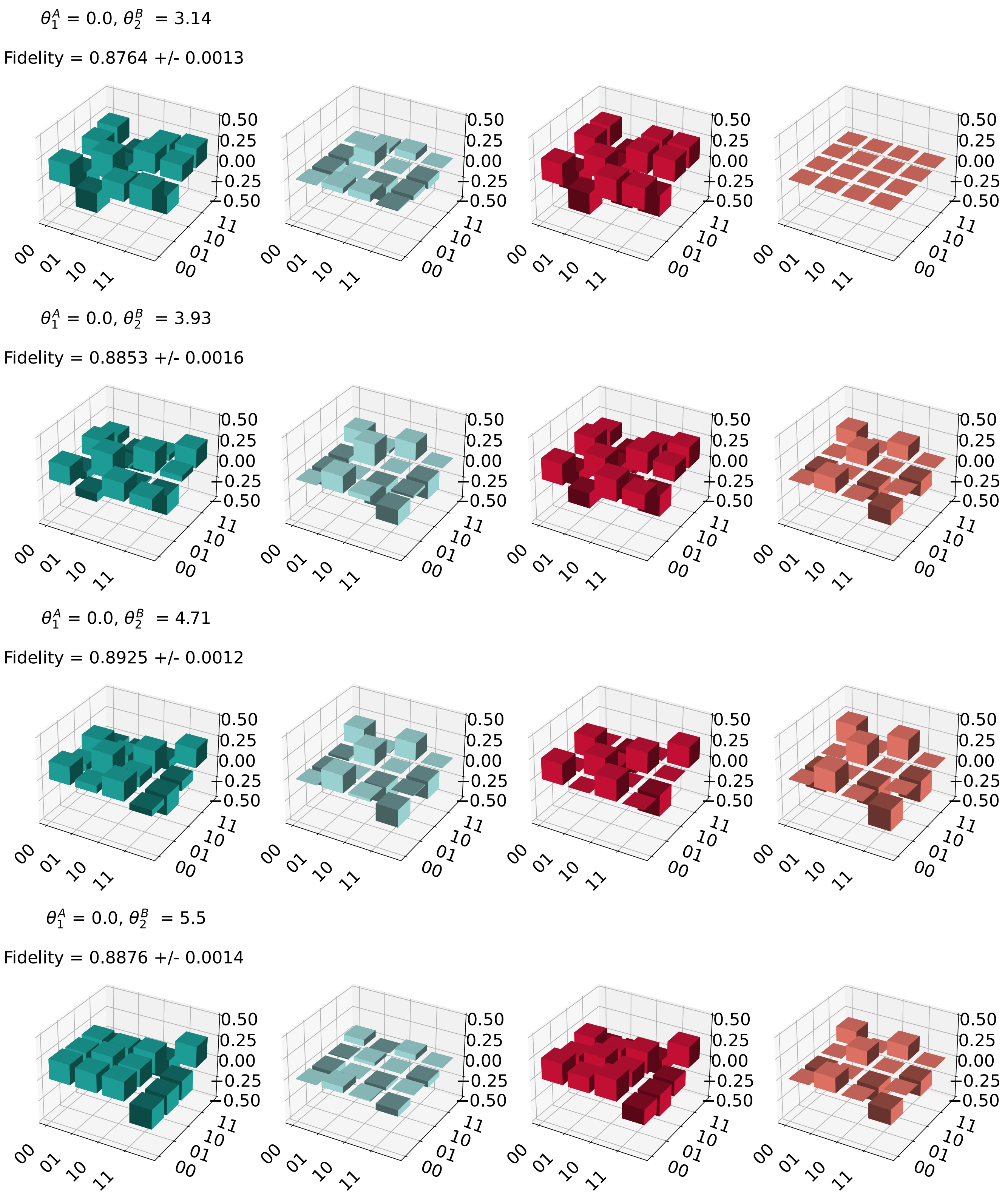
θ_1^A = 0.0, θ_2^B = 3.14
Fidelity = 0.8764 +/- 0.0013
θ_1^A = 0.0, θ_2^B = 3.93
Fidelity = 0.8853 +/- 0.0016
θ_1^A = 0.0, θ_2^B = 4.71
Fidelity = 0.8925 +/- 0.0012
θ_1^A = 0.0, θ_2^B = 5.5
Fidelity = 0.8876 +/- 0.0014
0.50
0.25
0.00
−0.25
−0.50
00
01
10
11

θ_1^A = 0.79, θ_2^B = 0.0

Fidelity = 0.8739 +/- 0.0013

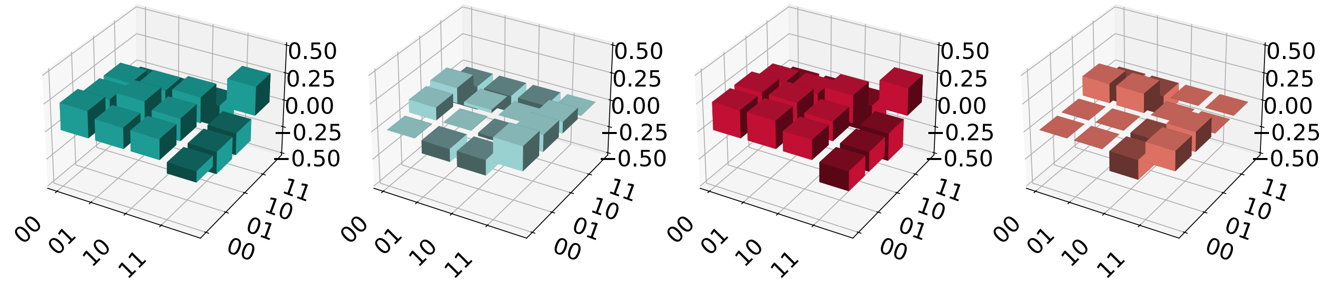

θ_1^A = 0.79, θ_2^B = 0.79

Fidelity = 0.9059 +/- 0.0017

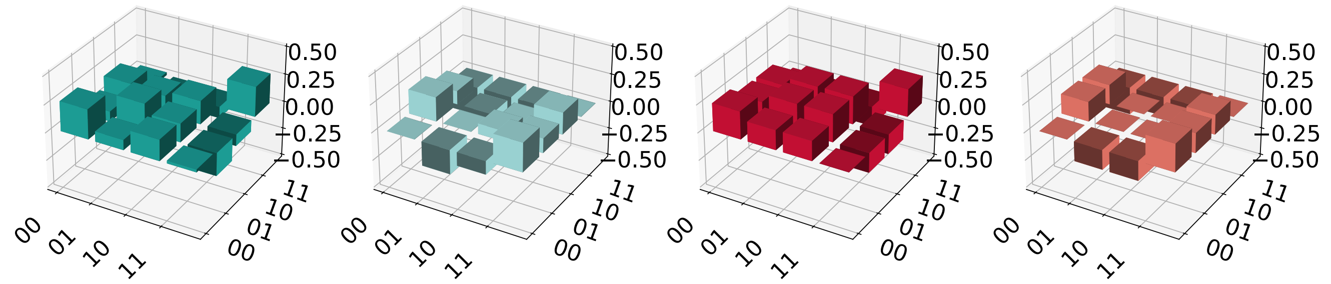

θ_1^A = 0.79, θ_2^B = 1.57

Fidelity = 0.8795 +/- 0.0015

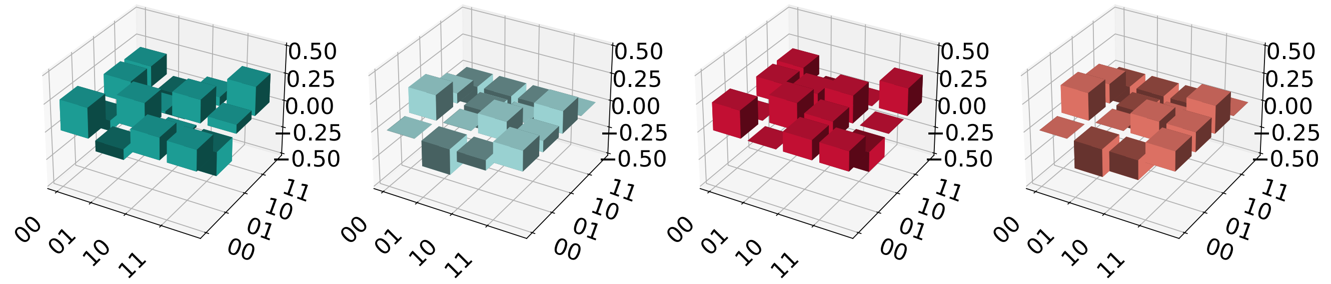

θ_1^A = 0.79, θ_2^B = 2.36

Fidelity = 0.8559 +/- 0.0016

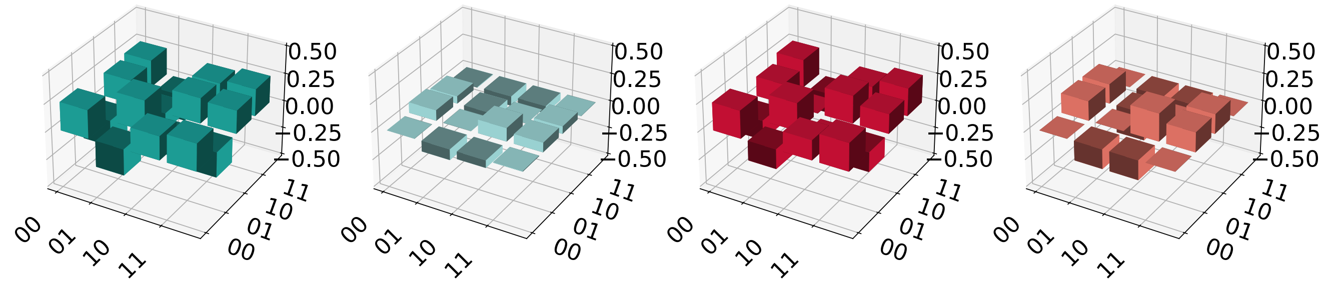

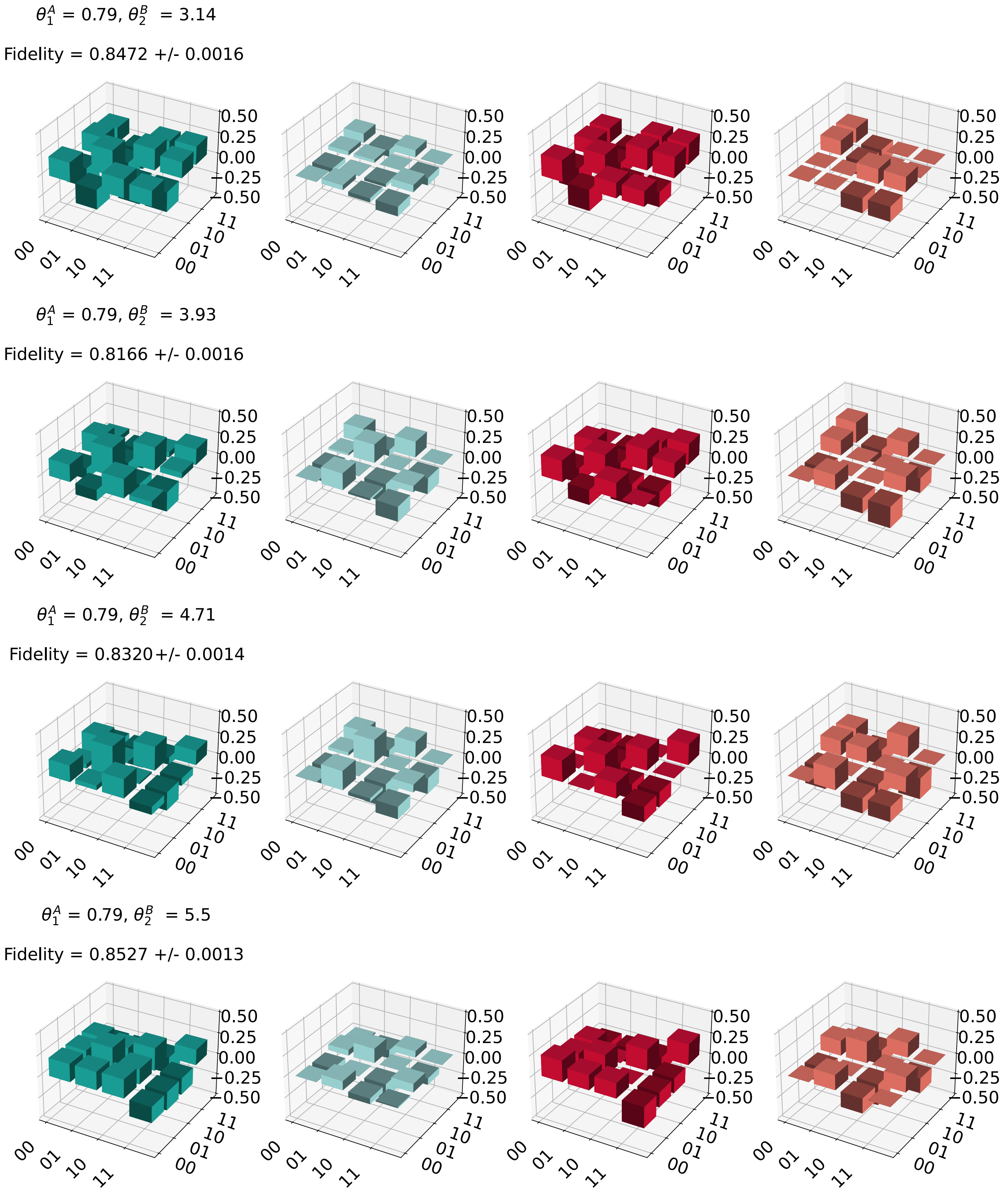

Supplementary Figure 5: **Experimental and ideal quantum state tomographies of** 16 **different initial two-qubit states.** We show in blue and light blue, respectively, the real and imaginary parts of the experimental density matrix, and in red and light red the real and imaginary parts of the ideal density matrix. We also report the corresponding fidelities.

SUPPLEMENTARY NOTE 8 - NOISE MODEL

Ideally, the state generated by the source is an entangled state in the form:

$$|\psi^-\rangle = \frac{1}{2}\left(|HH\rangle + |HV\rangle + |VH\rangle - |VV\rangle\right) \quad (5)$$

We modeled the experimental state by introducing white and colored noise, according to the following expression:

$$\rho_{noisy} = v\,|\psi^-\rangle\langle\psi^-| + (1-v)\left[\frac{\lambda}{2}\left(|\psi^+\rangle\langle\psi^+| + |\psi^-\rangle\langle\psi^-|\right) + \frac{1-\lambda}{4}\mathbb{I}_4\right] \quad (6)$$

where v is the visibility of the state and l is the fraction of coloured noise and $|\psi^+\rangle = \frac{1}{2}\left(|HH\rangle + |HV\rangle - |VH\rangle + |VV\rangle\right)$.

The parameters v and λ compatible with our data are $v \approx 0.76$ and $\lambda \approx 0.63$, on average for all states generated by varying the liquid crystals.

We also consider the imperfect phase shift inserted by the Pockels cell, which is on average $\approx -\pi/20$ with respect to the expected phase shift.

SUPPLEMENTARY NOTE 9 - CORRECTNESS OF THE PROTOCOL

Here, we report further details about the correctness of the protocol. In Supplementary Figure 6, we show the comparison between the average distance from the uniform distribution in the ideal, noisy-modeled case and experimental case for the algorithms considered in the Results section of the main text. We define the average distance between two distributions $\mathbf{p}$ and $\mathbf{q}$ as follows, where $p_i,\ q_i$ are the distribution elements and N is the total number of elements ($N = 4$ for our distributions):

$$d(\mathbf{p},\mathbf{q}) = \frac{\sum_i |p_i - q_i|}{N} \quad (7)$$

We chose as a figure of merit the distance from the uniform distribution since this is the distribution one would have if the computation resource was classical. Therefore, the plot in Supplementary Figure 6 provides additional validation of the nonclassicality of the computational resource.

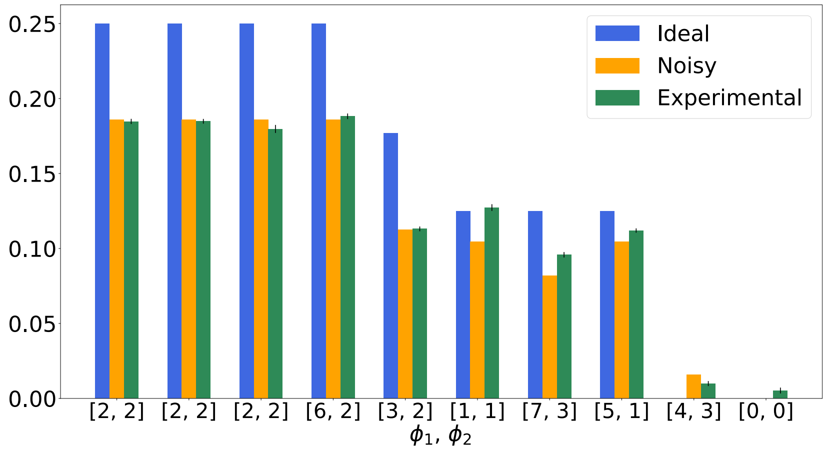

Supplementary Figure 6: **Expected average distance from the uniform distribution in the ideal, noisy-modeled, and experimental case**. In this bar plot, we show the expected average distance from the uniform distribution in the ideal case, in a model including experimental noise, and in the experimental case, for the algorithms considered in the main text. Uncertainties on the experimental data were estimated by assuming Poissonian statistics. Black bars correspond to one standard deviation.

In Supplementary Figure 7, we show that, by comparing each experimental distribution with each noisy-modeled one for all algorithms, the best overlap is with their noisy-modeled counterparts. This means that each algorithm can be distinguished from the others within a distance ≈ 0.05.

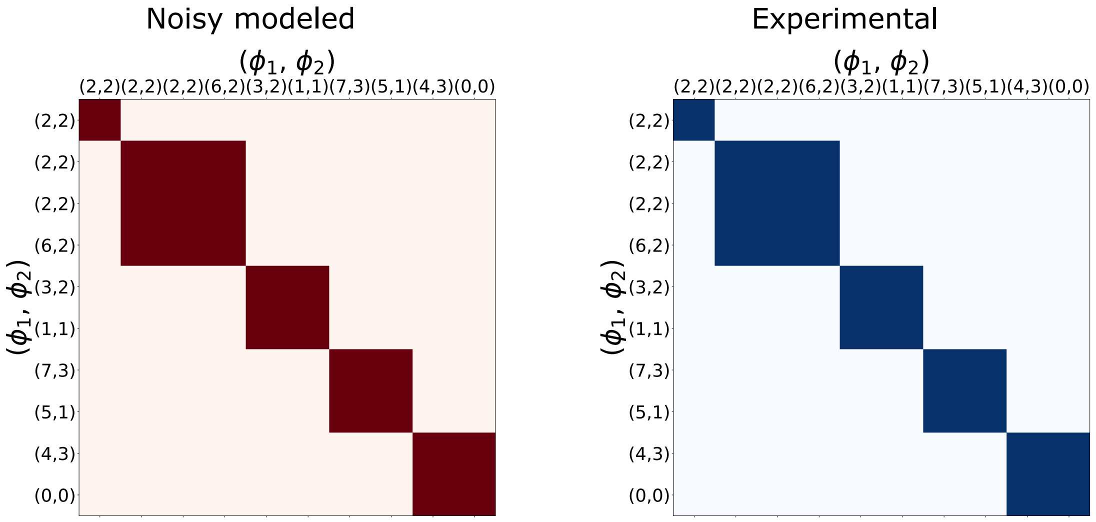

Supplementary Figure 7: **Average distance between noisy-modeled and experimental frequencies**. In these color matrices, we analyze the expected and measured overlaps between the algorithms considered in this work. In particular, in the red plot, we show the expected distances in the noisy model, while, in the blue one, we show the distance between experimental and noisy modeled frequencies. We highlight in dark color the cases in which the average distance between the distributions on the x-axis and y-axis is lower than or equal to 0.05. The light-colored parts, instead, correspond to distances greater than 0.05. The pairs (ϕ_1, ϕ_2) are indicated as integer multiples of $\frac{\pi}{4}$.

SUPPLEMENTARY REFERENCES

[1] M. Doosti, L. Hanouz, A. Marin, E. Kashefi, and M. Kaplan, "Establishing shared secret keys on quantum line networks: protocol and security," *arXiv preprint arXiv:2304.01881*, 2023.

[2] T. Kapourniotis, E. Kashefi, D. Leichtle, L. Music, and H. Ollivier, "Asymmetric quantum secure multi-party computation with weak clients against dishonest majority," *arXiv preprint arXiv:2303.08865*, 2023.

[3] Y. Ma, E. Kashefi, M. Arapinis, K. Chakraborty, and M. Kaplan, "QEnclave – a practical solution for secure quantum cloud computing," *npj Quantum Information*, vol. 8, no. 1, pp. 1–10, 2022.

[4] U. Maurer and R. Renner, "Abstract cryptography," in *Innovations in Computer Science*, pp. 1–21, Tsinghua University Press, jan 2011.

[5] V. Dunjko, J. F. Fitzsimons, C. Portmann, and R. Renner, "Composable security of delegated quantum computation," in *Advances in Cryptology – ASIACRYPT 2014* (P. Sarkar and T. Iwata, eds.), (Berlin, Heidelberg), pp. 406–425, Springer Berlin Heidelberg, 2014.

[6] A. Broadbent, J. Fitzsimons, and E. Kashefi, "Universal blind quantum computation," in *2009 50th Annual IEEE Symposium on Foundations of Computer Science*, pp. 517–526, IEEE, 2009.
